# The mistreatment of women during maternity care and its association with the maternal continuum of care in health facilities

**DOI:** 10.1186/s12884-024-06310-8

**Published:** 2024-02-13

**Authors:** Habtamu Kasaye, Vanessa Scarf, Annabel Sheehy, Kathleen Baird

**Affiliations:** 1 Collective for Midwifery, Child and Family Health, Faculty of Health, University of Technology Sydney, Sydney, NSW Australia; 2 Department of Midwifery, Institute of Health Sciences, Wollega University, Nekemte, Ethiopia

**Keywords:** Mistreatment of women, Maternity care, Health care facilities, Respectful maternity care, Continuum of maternity care

## Abstract

**Background:**

Mistreatment of childbearing women continues despite global attention to respectful care. In Ethiopia, although there have been reports of mistreatment of women during maternity care, the influence of this mistreatment on the continuum of maternity care remains unclear. In this paper, we report the prevalence of mistreatment of women from various dimensions, factors related to mistreatment and also its association to the continuum of maternity care in health facilities.

**Methods:**

We conducted an institution-based cross-sectional survey among women who gave birth within three months before the data collection period in Western Ethiopia. A total of 760 women participated in a survey conducted face-to-face at five health facilities during child immunization visits. Using a validated survey tool, we assessed mistreatment in four categories and employed a mixed-effects logistic regression model to identify its predictors and its association with the continuum of maternity care, presenting results as adjusted odds ratios (AORs) with their 95% confidence intervals (CIs).

**Results:**

Over a third of women (37.4%) experienced interpersonal abuse, 29.9% received substandard care, 50.9% had poor interactions with healthcare providers, and 6.2% faced health system constraints. The odds of mistreatment were higher among women from the lowest economic status, gave birth vaginally and those who encountered complications during pregnancy or birth, while having a companion of choice during maternity care was associated to reduced odds of mistreatment by 42% (AOR = 0.58, 95% CI: [0.42–0.81]). Women who experienced physical abuse, verbal abuse, stigma, or discrimination during maternity care had a significantly reduced likelihood of completing the continuum of care, with their odds decreased by half compared to those who did not face such interpersonal abuse (AOR = 0.49, 95% CI: [0.29–0.83]).

**Conclusions:**

Mistreatment of women was found to be a pervasive problem that extends beyond labour and birth, it negatively affects upon maternal continuum of care. Addressing this issue requires an effort to prevent mistreatment through attitude and value transformation trainings. Such interventions should align with a system level actions, including enforcing respectful care as a competency, enhancing health centre functionality, improving the referral system, and influencing communities to demand respectful care.

## Introduction

The continuum of maternal, neonatal, and childcare is widely recognised as a critical intervention to reduce maternal, neonatal, and child mortality [1]. The provision of quality care plays a pivotal role in ensuring the continuity of these essential services. A woman's humiliating interaction with her health care provider (HCP) or other staff members, as well as the state of the health infrastructure and systems, are all considered detrimental to the quality of maternity care [2]. Various terminologies have been used to describe such experiences of women, including disrespect and abuse [3], maltreatment [4], and obstetric violence [5], underscoring the importance of ensuring terminological clarity. To focus on woman's experience and minimise individual blame [6], in this study, we refer to the mistreatment of women during maternal health care as defined in the methods section.

Despite the World Health Organization's (WHO) declaration affirming every woman's right to receive the highest standard of care with dignity and respect [7], mistreatment of childbearing women remains a pervasive global public health issue reported across all nations and cultures [7]. In Sub-Saharan Africa (SSA), the pooled prevalence of mistreatment was reported to affect up to 44% of women [8]. Mistreatment of childbearing women is not only confined to low- and middle-income countries; it is also reported in high-income countries with resilient health care resources. One out of every five women in Europe [9], one-sixth of women in the United States of America [10], two out of every five women in Latin America [11], and one in every ten women in Australia [12] have experienced mistreatment.

In Ethiopia, various forms of disrespect and abuse categories discussed by Bowser and Hill [3] were reported from different regions [9]. The prevalence of these mistreatment forms ranges from 14.5% when reported by healthcare providers [10] to almost all women self-reporting (98.9%) [13] experiencing at least one form of mistreatment. Most studies conducted in Ethiopia have focused on the mistreatment of women, as reported by women. However, these studies often conflate various categories of mistreatment without considering their dimensionality [13–22]. While such aggregation of various categories of mistreatment is important for understanding the issue, addressing them separately is crucial for effective and focused interventions in the actual context.

When women experience mistreatment in health care facilities during their pregnancy or birth, it continues to have a negative impact on their lives. It can decrease women's confidence in health facilities which can lead to an increased likelihood of maternal complications due to a lack of engagement in services [23–25]. Furthermore, it can be a deterrent to service utilisation, even more than sociocultural beliefs and practice [26]. Due to a history of mistreatment, it is alleged women in low-income countries regardless of pregnancy risk factors would prefer to give birth at home with dignity, alone or with a family member, than face mistreatment in a health facility [2, 27].

Such challenge becomes even more significant when these mistreatments occur in countries where maternal continuum of care dropouts are common, as is the case in Ethiopia. This is particularly pertinent in Ethiopia, where maternal care discontinuation is a pressing concern. The 2019 Ethiopian Mini Demographic and Health Survey revealed a significant gap in continuum of maternal care. While 74% of women started antenatal care, only 48% gave birth in health care facilities, and merely 34% received postnatal checks within two days of the birth [28]. This signals significant problem with service discontinuation and calls for an investigation into the underlying factors.

Previous studies have identified various socio-demographic and contextual factors associated with the completion of continuum of maternity care [29, 30]. However, there is limited evidence linking experiences of mistreatment and its impact on the extent of continuity of care. To fully understand the link between mistreatment and continuum of care, it is essential to measure mistreatment of women using a validated tools focusing on specific concepts at a time. Therefore, the aim of this study was to determine the prevalence of mistreatment of women in various dimensions, identify the factors associated with mistreatment, and identify its association with the continuum of maternity care in health facilities.

## Methods

### Study design and setting

A cross-sectional survey-based study was conducted from February to June 2022, adhering to the STROBE reporting guideline. This paper is part of larger mixed method study conducted in western part of Ethiopia, East Wollega Zone Oromia regional state. Ethiopia is administratively divided into twelve regions and two city administrations. These regions have been further subdivided into zones, woredas or districts, and kebeles (the lowest tier of government administrative units). Based on the 2007 Ethiopian population and housing census projections, the average projected population of Ethiopia in 2022 was 105.17 million, and about 78% of the population lived in rural areas [31].

Ethiopian health service delivery is structured in a three-tier system of primary, secondary, and tertiary levels. The primary level, or primary health care (PHC) unit, comprises health posts, health centres, and a primary hospital that provides services for about 100,000 people. It provides blood transfusion and emergency surgical services, including caesarean sections. The secondary level is a general hospital, which serves as a centre of health care for an average of one million people and is a referral centre for primary hospitals. A referral centre for the general hospital is the tertiary level, a specialised hospital providing care for an average of five million people [32, 33].

East Wollega zone, where this study was conducted, is one of administrative zones of Oromia Regional State whose capital city is Nekemte and contains one special-city woreda (Nekemte), 17 woredas, 43 towns and 287 rural kebeles. The East Wollega zone's total population was 1.806 million in 2022. The number of women of reproductive age accounts for 18% of the total population with a total fertility rate of 4.07. East Wollega zone has two tertiary hospitals, both found in Nekemte town, three district hospitals and 67 health centres [31–33].

### Study participants

The study population includes women who received at least one component of the continuum of maternity care services (antenatal care, labour and birth or postnatal care) and gave birth within three months before the data collection period. Hence, study participants were women who had given birth within the past three months after accessing maternal health care services from health facilities. They were approached between six weeks and three months after giving birth to capture their experiences related to mistreatment and service utilization throughout the continuum of care, covering the antenatal period, childbirth, and the postnatal period.

To minimise bias, women were excluded from the study if they had a personal relationship with any person employed at any of the health care facilities where recruitment occurred. Women who were unable to provide consent, had experienced a recent stillbirth, or were at risk of psychological trauma were also excluded from the study.

### Sampling and recruitment

The total sample of women included in the survey was calculated using a single population proportion formula in which the proportion (prevalence) of the mistreatment of women was 67.1% (from study conducted in Northern Ethiopia by Wassihun et al. [21]), and a 95% confidence level, with a 5% margin of error, and the cluster effect of 2, the desired sample size was 679. After adjusting the desired sample size for a 10% non-response rate, the total sample size for women participants was determined to be 755. To enhance the robustness of the findings, 760 participants which gives the power 90% were approached in the study.

For logistical and safety reasons related to the civil conflict in Ethiopia, participants were purposively selected from four woredas: Diga, Nekemte, Guto gidda and Sasiga. Two hospitals, three health centres and one health post were selected to invite women to participate in the study. The women were approached to participate when they visited the health facilities to have their child vaccinated. This opportunity for recruitment into the study was chosen as 97% of all mothers visit a health facility to have their children vaccinated [33]. Utilising the vaccination clinics also reduced any accessibility difficulties the researcher may have encountered due to some of the geographical locations which are widespread and difficult access especially during a time of civil unrest. All the women who came to the vaccination clinic with their newborn baby and who met the inclusion criteria were approached to participate in the study between February and June 2022.

### Measurements

#### Outcome variables

In this study, we focused not only on mistreatment of women during childbirth but also during antenatal care and postnatal checkups and their link with continuum of care. Hence, under the aims assessing magnitude of mistreatment of women and associated factors, mistreatment of women is outcome variable and then it is the exposure variable for the model that assessed its association with continuum of care. To achieve these, various categories of mistreatments of women were measured under two broader dimensions—the overt and covert as discussed by Bohren et al. [34]. The overt mistreatment results from outright intentional abuse against women (physical abuse, verbal abuse, stigmatisation and discrimination) that parallels with a broader violence against women framework [35]. We categorised the mistreatment arising from such intentional abuses under the latent construct of interpersonal abuse. In other hand, the latent construct underlying covert mistreatment includes failure to maintain professional standards of care, the poor rapport between a woman and the health care provider, as well as any constraints related to health facilities and system limitations which occurs during the process of the care and are related to the broader quality of care framework [36].

The maternal continuum of care encompasses the care provided to women during pregnancy, birth, and the postnatal period. Although completion of continuum of care should include all components of these three stages, for the purpose of analysing the association between mistreatment and the continuum of care, a woman was considered to have completed the continuum of care if she received four or more episodes of antenatal care, a skilled health care provider attended the birth of the baby and, she received at least two episodes of postnatal care. The first episode of postnatal care was expected to be provided within 24 h of birth, and the next between 48- and 72-h following birth.

#### Primary exposure variable

Various forms of mistreatment including interpersonal abuse, failure to maintain professional standards of care, poor rapport between a woman and the health care provider, and health system conditions and constraints were exposure variables for the analysis conducted to assess the association between mistreatment and maternal continuum of care.

#### Independent variables

- Sociodemographic characteristics: woman’s age, residence (urban–rural), educational status, average monthly income- measured as a factor variable (lowest, low, middle, and highest) based on the income quintile.
- Pregnancy and birth: parity, mode of birth, support from a partner or household members, complication during pregnancy or childbirth.
- The quality of antenatal care: was based on the proportions of components of care received as recommended by WHO [37] including: recording of blood pressure, received tetanus toxoid immunisation, iron folate, advice on complications readiness (advice on danger signs like occurrences of severe headache, convulsion, blurring of vision, vaginal bleeding, upper abdominal pain, sudden gush of fluid per vagina, decreased fetal movement, lower abdominal tenderness and foul smelling vaginal discharge), birth preparedness, nutrition counselling, blood sample for basic investigations such as blood group and Rh factor, haemoglobin, screening test for syphilis, Hepatitis B virus surface antigen test and HIV testing, urine testing and drugs for intestinal parasites. For each of these care components, a code of 1 was assigned if the women reported receiving the specific component and 0 if not. The total sum score of these ten items was then calculated to represent the quality of antenatal care received.
- Health service-related factors: type of the health facility, primary care providers, presence of companion, date, and time of birth.

### Data collection instruments and process

A validated community-based survey tool developed by a WHO-led study [6] was adapted and translated in to Afaan Oromo to measure the level of mistreatment of women during maternity care. This tool was developed based on a mixed-method and iterative approach. A total of 32 items were used to measure the level of mistreatment of women based on seven typologies as identified by Bohren et al. [34].

Two data collectors were recruited for the data collection. The data collectors did not have an employment background in health sciences; they were engaged only to assist women with low literacy levels to complete the survey. Before they were able to assist with survey data collection, training regarding the objective of the study, data collection instrument, data collection techniques and methods was provided by the researcher to minimise their influence on participant responses. Survey data was collected through a paper-based interviewer administered questionnaire. Each woman was asked individually about their sociodemographic, pregnancy and birth related characteristics, and the care received and experiences during their recent childbirth in the health facilities where they received maternity care (antenatal care to postnatal care).

### Data analysis

Survey data were checked for completeness and entered Epi-info version 7.1 software for data entry based on a predesigned data entry template. The data entered to Epi-info were exported to Stata version 17 for cleaning and analysis [38]. Descriptive analysis of sociodemographic, pregnancy, birth and postnatal characteristics were performed. These descriptive statistics were calculated and presented using tables, figures, and text: median and interquartile range for the variables which were continuous but not normally distributed, and frequencies and percentages for categorical variables were reported.

Thirteen indicators of mistreatment were included under interpersonal abuse. Six of these indicators focused on physical abuse which included being hit, slapped, or pinched, gagged, tied to the bed, forcefully held down, denial of pain relief, and forceful abdominal/fundal pressure. On the other hand, verbal abuse included being shouted, screamed, insulted, scolded, mocked, hissed at, receiving negative comments related to the woman’s status, being threatened and blamed. Discriminatory comments related to woman’s educational status, age, language, or economic status, feeling discriminated against in any way and held back from asking concerns were indicators included under stigma and discrimination category. Women who experienced at least one category of physical abuse, verbal abuse or stigma and discrimination was coded as ‘Yes = 1’, while those who did not experience were coded as ‘No = 0’.

A mixed-effect logistic regression model was fitted to identify factors associated to interpersonal abuse. The model was specified using the generalized linear mixed effects regression (*glmer*) function of *lme4* package [39] using R Statistical Software [40]. Variables significantly associated to outcome variable at *p*-value less than 0.2 in multilevel bivariable analysis were taken as candidates in multi-variable analysis. To obtain the best fitted model, three models were constructed: the first model being intercept-only model with five health facilities being a random effect variable; the second model with fixed effects for individual factors related to sociodemographic, pregnancy and birth related characteristics of the women and random effect for health facilities; and the third model containing contextual variables in addition to variables included in model two as fixed effects and health facilities for random effect variable.

To model the association between mistreatment of women and completion of continuum of care, three multilevel mixed-effects models were employed using same function and package discussed above. The first model served as a null model with only an intercept, the second model included random intercepts for health facilities, and the fixed effects consisted of interpersonal abuses and experiences of poor rapport between women and healthcare providers. The final model, which is a better fitted model, incorporated additional socio-demographic, pregnancy, and childbirth-related variables to assess the independent association between mistreatment and the continuum of care. Here, the outcome variable was completion of continuum of maternity care, i.e., having at least four antenatal care check-ups, birth attended by skilled care providers and at least two postnatal check-ups within first week of birth.

Setting health facilities as a random-effect variable in all models allowed us to account for the correlation between individuals who received care from the same health facility. By this approach we were able to obtain more precise estimates of the fixed effects of predictors and effectively control for the clustering effect, as recommended by Faraway [41]. The Akaike information criterion (AIC) and Bayesian information criterion (BIC) was used to assess the model fit. Predictor variables having multi-collinearity (correlation) to each other were checked by variance inflation factors (VIF) and removed from the final model. Each model's effect sizes were reported as adjusted odds ratios (AOR) with their 95% confidence intervals (CI).

## Results

### Sociodemographic characteristics of the participants

Overall, 760 women completed the survey, and all received their maternity care from health facilities. The participants ages ranged from 18 – 39 years with a median age of 25 years and interquartile range (IQR) of 5. The majority (*n* = 735; 96.7%) were married. Three out of four women (*n* = 576; 75.79%) were of a Protestant religion and most participants (*n* = 743; 97.76%) identified themselves as Oromo in ethnicity. One in every five women were unemployed. Most of the participants resided in urban and semiurban areas (*n* = 662; 87.11%). Three hundred thirty-eight (44.47%) women were educated to college diploma or university degree level, while nearly half (*n* = 392; 51.58%) of their husbands/partners were educated to college and above level. The median average monthly income of the women's household was 3000 Ethiopian birr (ETB) with an IQR of 3000 ETB, and three-fourths of them earned less than 5000 ETB. The median travel time to the nearest health facility was reported to be five minutes by car (median 5 ± 7.5) (Table 1).

**Table 1:** Sociodemographic characteristics of women participated in survey, East Wollega Zone, Western Ethiopia, 2022 (*n* = 760)

Characteristics	Categories	Frequency	Percentage
**Age (years)**	18–19	37	4.9
	20–24	339	44.6
	25–29	279	36.7
	30–34	80	10.5
	35 +	25	3.3
**Marital status**	Married	735	96.7
	Others^a^	25	3.3
**Religion**	Protestant	576	75.8
	Orthodox	142	18.7
	Muslim	40	5.3
	Others	6	0.8
**Ethnicity**	Oromo	743	97.8
	Amhara	13	1.7
	Others	4	0.5
**Occupation**	Government employee	171	22.5
	Housewife	165	21.7
	Private business	154	20.3
	Agriculture	83	10.9
	Unemployed	158	20.8
	Student	29	3.8
**Residence**	Rural	98	12.9
	Urban	662	87.1
**Educational status**	No formal education	35	4.6
	Primary school	159	20.9
	Secondary school	228	30.0
	College and above	338	44.5
**Husband educational status**	No formal education	27	3.5
	Primary school	121	15.9
	Secondary school	220	28.9
	College and above	392	51.6
**Income (Median and IQR = 3000)**	Lowest (< 2000 ETB)	186	24.5
	Low (2000-3000ETB)	163	21.5
	Middle (3000–5000)	228	30.0
	Highest (> 5000)	183	24.1
**Travel distance to the health facility by vehicle** **Median 5 and IQR = 7.5**	< 2.5 min	243	32.0
	2.5-5min	181	23.8
	5-10min	165	21.7
	10 and above	171	22.5

^a^Others-includes single, divorced, widowed, cohabit and separated individuals

### Pregnancy, birth, and postpartum related characteristics of the participants

Most of the participants’ pregnancies were planned (*n* = 673; 88.5%) and the majority of women were supported during their pregnancy and birth (*n* = 744; 97.9%) by the woman’s partner and other relatives. Two out of every five women were primiparous (*n* = 310; 40.8%), and the typical number of previous births among women were two.

Fifty-one women (6.7%) reported having an earlier stillbirth, while 10.0% (*n* = 76) reported experiencing complications during their last pregnancy. Of these women, 31.6% (*n* = 24) experienced a hypertensive disorder of pregnancy, while others reported antepartum haemorrhage (13.2%), premature rupture of membranes (19.7%), and postpartum haemorrhage (6.6%) among other complications included anaemia, hyperemesis gravidarum, oligohydramnios and Rh- isoimmunisation (Table 2).

**Table 2:** Pregnancy, birth, postpartum care related characteristics of women, East Wollega Zone, Western Ethiopia, 2022 (*n* = 760)

Characteristics	Categories	Frequency	Percentage
**Pregnancy plan**	Yes	673	88.5
**Pregnancy was supported**	Yes	744	97.9
**Number of births (parity)** **Median 2 ± 2**	Primiparous	310	40.8
	Multiparous	409	53.8
	Grand multiparous	41	5.4
**Previous history of stillbirth**	Yes	51	6.7
**Pregnancy complication**	Yes	76	10.0
**Causes of pregnancy complications (*n *= 76)**	Hypertensive disorder	24	31.6
	Antepartum haemorrhage	10	13.2
	Premature rupture of membrane	15	19.7
	Others antepartum complications^a^	22	28.9
	Postpartum haemorrhage	5	6.6
**Received antenatal care**	Yes	759	99.9
**Health facility where antenatal care received (*n *= 759)**	Health centre	359	47.3
	Hospital	380	50.1
	Private clinic	17	2.2
	Health post	3	0.4
**Gestational age at first ANC (weeks) (*n *= 759)** **Median 16 ± 8**	First trimester	305	40.2
	Second trimester	438	57.7
	Third trimester	16	2.1
**Number of antenatal cares received (*n* = 759)**	One-three	260	34.3
	Four above	499	65.7
**Place of birth**	Health centre	116	15.3
	Hospital	596	78.4
	Private clinic	13	1.7
	Home	34	4.5
	On the way to health centre	1	
**Skilled assistance at birth (SAB)**	Yes	725	95.4
	Four + ANC and SAB	484	63.7
**Mode of birth**	Spontaneous vaginal birth	564	74.2
	Operative vaginal birth	92	12.1
	Caesarean section	104	13.7
**Time of birth**	Day	478	62.9
	Night	282	37.1
**Day of birth**	Weekday	477	62.8
	Weekend	283	37.2
**Postnatal care (PNC)**	PNC within 24 h of birth	719	94.6
	PNC on the third day of birth to seven days	136	17.9
	PNC between 7–14 days of birth	27	3.5
	PNC on six weeks of birth	5	0.7

^a^Anaemia, hyperemesis gravidarum, oligohydramnios, Rh- isoimmunisation

**Fig. 1:**
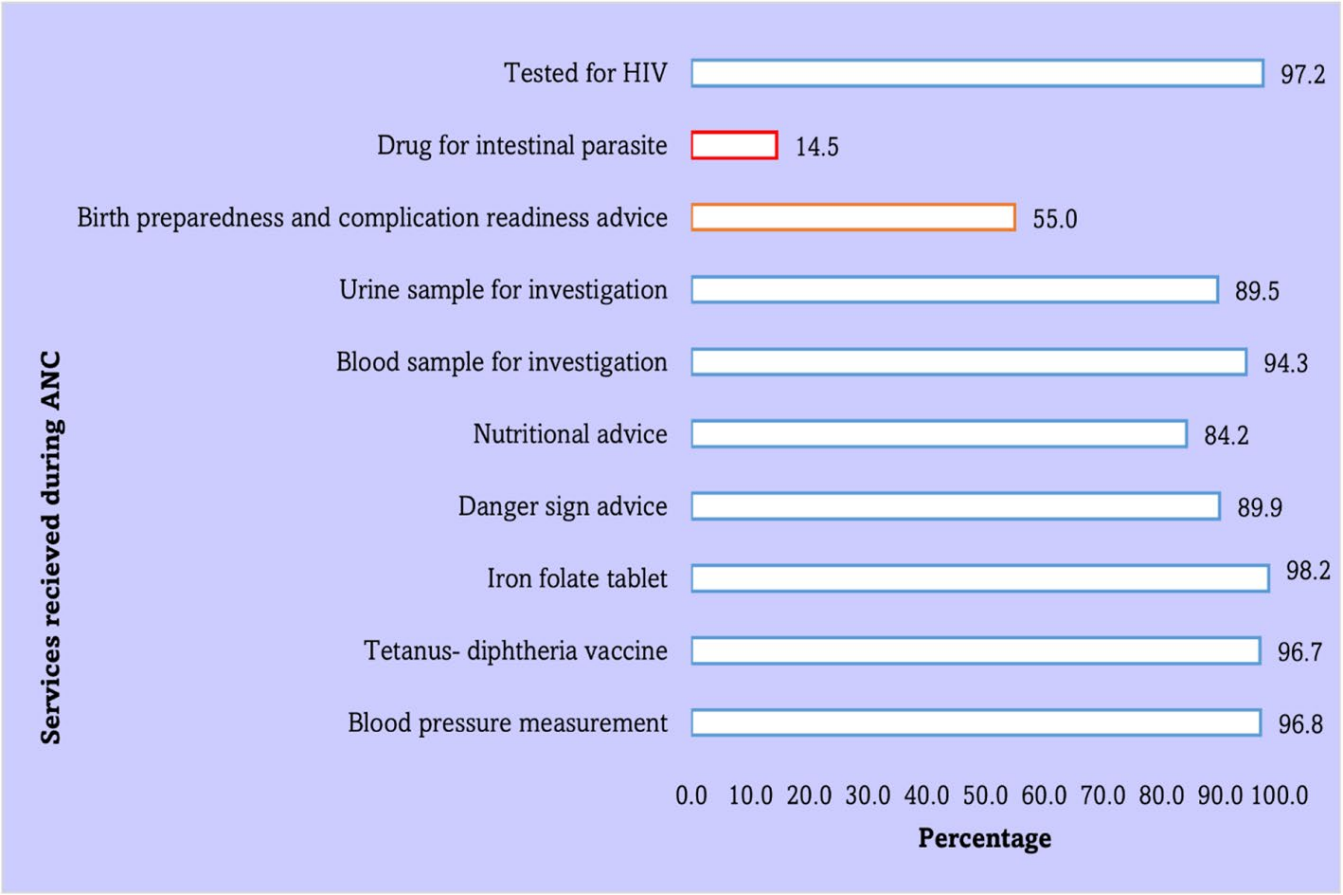
Bar chart showing the percentage of services given to women attending antenatal care from health facilities of East Wollega Zone, Western Ethiopia, 2022 (*n* = 759)

Figure 1 depicts the services women received from health facilities during their antenatal care. Most women received eight of ten routine components of care, however less than one-sixth (*n* = 110; 14.5%) were given drugs for intestinal parasite.

### Continuum of maternity care

Almost all women (*n* = 759; 99.9%) attended at least one antenatal care visit at health facilities. Of these, 380 (50.1%) received prenatal care at public hospitals while 359 (47.3%) received it at health centres. Only two out of five women (*n* = 305; 40.1%) began to receive antenatal care during the first trimester of pregnancy as recommended by the WHO (2016) antenatal care guideline most of the women received their first antenatal care visit at 16 weeks gestation.

Among the women who received antenatal care, two-thirds (*n* = 499; 65.7%) received four or more antenatal care episodes while 97.0% of these women and 63.7% (*n* = 484) of women all women received four and above antenatal care check-ups and their birth were also attended by skilled providers. From which 136, 17.9% of all women completed continuum of care up until second post-natal check-ups within first week of birth. Only three women received all components of continuum of maternity care, including four or more antenatal care visits, skilled health provider attendance during birth, and postnatal care up to six weeks after birth.

**Fig. 2:**
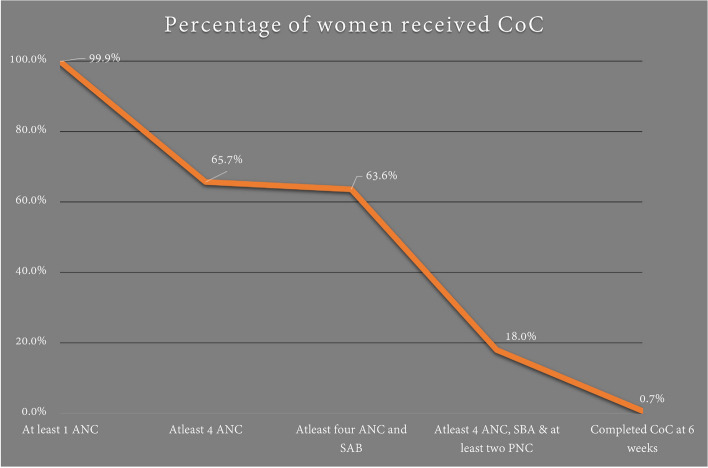
Percentage of women received continuum of maternity care from health facilities (initial ANC to six week postnatal) of East Wollega Zone, Western Ethiopia, 2022 (*n* = 759)

Skilled health care providers including midwives, nurses and medical doctors assisted with 725 (95.4%) births. Most of the births (*n* = 596; 78.4%) occurred in public hospitals, while 34 (4.5%) women gave birth at home. Almost three-quarters of births (*n* = 564; 74.2%) were a spontaneous vaginal birth (Table 2 and Fig. 2).

### Mistreatment of women

#### Interpersonal abuse

This category of mistreatment occurred during direct interactions between the woman and health care providers and includes any form of physical and /or verbal abuse, or stigma or discrimination. A total of 284 (37.4%, 95% CI: [34.32–41.23]) women reported experiencing at least one form of interpersonal abuse. Most of the abuse (*n* = 248; 87.3%) occurred during woman’s labour and birth, while some women also experienced mistreatment during their antenatal or postnatal care check-ups. Fourteen (4.9%) women reported experiencing mistreatment in more than two care units, including antenatal care, during birth, and postnatal care (Table 3).

**Table 3:** Mistreatment that women experienced during maternity care in health facilities of East Wollega Zone, Ethiopia, 2022 (*n* = 753)

Categories of mistreatment	Sub-categories	Frequency (%)
**Interpersonal abuse**	**Any physical abuse, verbal abuse, stigma, or discrimination**	**284(37.4%)**
**Physical abuse**	**Any physical abuse**	207(27.2%)
	Denial of pain relief while in health facility	142(18.7%)
	Forceful abdominal downward pressure (fundal pressure)	77(10.1%)
	Hit or slapped or pinching or pinched by materials	70(9.2%)
	Gagged	20(2.6%)
	Forcefully held down to the bed	12(1.6%)
	Tied to the bed	3(0.4%)
**Verbal abuse**	Any verbal abuse	158(20.8%)
	Shouted, screamed, insulted, scolded, mocked, hissed at	150(19.7%)
	Negative comments	28(3.7%)
	Threatened	15(2.0%)
	Blamed	14(1.8%)
**Stigma and discrimination**	Any discrimination	36(4.7%)
	Negative comments (educational status, age, tribe, language, or economic status…)	19(2.5%)
	Felt discriminated	22(2.9%)
	Held back from asking concerns	5(0.7%)
**Failure to meet professional standard**	Overall failure of professional standard of care	227(29.9%)
	Lack of consented care	190(25.0%)
	Lack of information	185(24.3%)
	Lack privacy	31(4.1%)
	Ignored or neglected	21(2.8%)
	Non-confidential care	15(2.0%)
	Felt nuisance	8(1.0%)
**Poor rapport with health care providers**	Overall poor rapport b/n health care providers and women	387(50.9%)
	Lack of birth companion	329(43.3%)
	Not allowed to move during labour	48(6.5)
	Emotionally unsupported	29(3.8%)
	Did not receive response for concerns /questions	17(2.2%)
	My concerns were not heard	14(1.8%)
	Not allowed to eat	6(0.8%)
**Health system conditions and constrains**	Overall health systems constrain	47(6.2%)
	Lack of bed during PNC	39(5.3%)
	Lack of bed during labour & birth	11(1.5%)
	Bribe/asked for informal payment or gift	4(0.5%)
	Ordered to clean up after oneself	1

Physical abuse was the most common form of interpersonal abuse overall, with 207 (27.2%) women reporting experiencing some form of physical abuse. The most reported types of physical abuse included denial of pain relief during procedures such as episiotomy and after caesarean section, which could also be considered emotional abuse (*n* = 142; 18.7%) followed by forceful abdominal pressure, known as fundal pressure (*n* = 77; 10.1%). Other types of physical abuse reported included slapping, punching, or pinching by either a finger or an object (*n* = 70; 9.2%) among others.

One hundred fifty-eight (20.8%) women reported experiencing verbal abuse, which included being shouted and screamed at, insulted, scolded, mocked, or hissed at. Verbal abuse included negative comments, blaming, and threats. Additionally, women reported experiencing provider prejudice based on their social status. Thirty-six (4.7%) women shared that they encountered negative comments from healthcare providers, which made them feel discriminated against or inferior. These negative experiences hindered their ability to express their concerns about themselves and/or their baby.

#### Failure to meet professional standard of care

Two hundred twenty-seven (29.9%) received substandard care, with healthcare providers failing to obtain their consent for certain procedures or exams and frequently withholding crucial information. One hundred-ninety (25.0%) women reported that they were not asked for consent nor were they offered an explanation or rationale for the health care provider performing certain procedures such as an abdominal or vaginal examination during labour and birth. Women also reported a lack of privacy (*n* = 31; 4.1%) when in the health facility and reported being neglected or ignored (*n* = 21; 2.8%) (see Table 3).

#### Poor rapport between women and health care providers and health systems constrains

Half of the women (*n* = 387; 50.9%) reported having an unsatisfactory interaction with health care providers. Three hundred twenty-nine (43.3%) women were not allowed to have companion of their choice present with them during antenatal care, labour, and birth or during immediate postpartum period in health facilities, leaving them feeling emotionally unsupported due to being ordered to remain still or a lack of response from healthcare providers.

Forty-seven (6.2%) women reported facing health system-related constrains regarding resources at the heath care facility, ranging from unavailability of a bed during a woman’s birth or immediate postnatal period. Indeed, 39 (5.3%) women reported having no access to a bed during their birth, and 11(1.5%) were unable to access a bed after birth, as presented in Table 3.

### Factors associated with mistreatment of women

When comparing the standard logistic regression to the null multilevel mixed-effect binary logistic regression model, we found evidence of variation in women's mistreatment experiences across healthcare facilities (likelihood ratio test: χ2 = 68.5, df = 14, *p* < 0.001). This suggests that the multilevel mixed-effects model is a better fit. The null model yielded an Intraclass Correlation Coefficient (ICC) of 6.4%, indicating that approximately 6.4% of mistreatment variability in can be attributed to differences between the health facilities (Table 4).

**Table 4:** Factors associated with mistreatment of women during maternity care in East Wollega Zone, Western Ethiopia, 2022 (*n* = 752)—results from Mixed-Effects Logistic Regression Models

**Variables**	**Model I** **AOR [95%CI]**	Model II AOR [95%CI]	Model III	
			AOR [95%CI]	*P*-values
**A. Fixed effects**				
**(Intercept)**	**0.40[0.25 – 0.65]**	**0.15 [0.04 – 0.53]**	**0.14 [0.04 – 0.52]**	**0.003**
**Woman’s age (years)**		1.03 [0.99 – 1.07]	1.03 [0.99 – 1.07]	0.140
**Educational status**				
**Highschool and below**		*Reference*	*Reference*	
**College and above**		0.88 [0.59 – 1.30]	0.86 [0.58 – 1.27]	0.440
**Socio-economic status**				
**Lowest**		1.84 [1.09 – 3.09]	1.80 [1.06 – 3.04]	0.029
**Low**		0.61 [0.35 – 1.05]	0.62 [0.36 – 1.06]	0.081
**Middle**		0.94 [0.60 – 1.49]	0.93 [0.58 – 1.47]	0.746
**High**		*Reference*	*Reference*	
**History of stillbirth**				
**No**		*Reference*	*Reference*	
**Yes**		0.50 [0.25 – 0.99]	0.49 [0.24 – 0.97]	0.041
**Presence of complication**				
**No**		Reference	*Reference*	
**Yes**		1.80 [1.06 – 3.04]	1.78 [1.05 – 3.01]	0.031
**Mode of birth**				
**Vaginal birth**		*Reference*	*Reference*	
**Instrumental**		1.41 [0.87 – 2.29]	1.54 [0.94 – 2.50]	0.085
**Caesarean section**		0.55 [0.34 – 0.90]	0.59 [0.36 – 0.98]	0.042
**Birth companion**				
**No**			*Reference*	
Yes			0.58 [0.42 – 0.81]	< 0.001
**Hospital versus health centre**				
**Health centre**			*Reference*	
**Hospital**			2.20 [0.87 – 5.56]	0.096
**B. Random effects**				
**Health facilities**				
**Variance**	0.223	0.31	0.197	-
**Intraclass correlation (ICC)**	0.064	0.087	0.057	-
**C. Model fitness**				
**AIC**	966	935	926	-
**BIC**	975	999	999	-
**Log-Likelihood**	-481	-453	-447	-
**Deviance**	962	907	894	-
***P*-value**	-	< 0.001	< 0.001	-

In the final multivariable multilevel mixed-effects model, several factors were found to be significantly associated with interpersonal abuses experienced by women in health facilities. These factors include the economic status of the women, the occurrence pregnancy or birth complications, previous history of stillbirth, mode of birth, and the presence of a companion during maternity care (Table 4).

Women from different economic statuses experienced mistreatment to different extents. Specifically, women from the lowest economic status had 80% higher odds of experiencing mistreatment compared to those from higher economic backgrounds (AOR = 1.80, 95% CI: [1.06 – 3.04], *p*-value = 0.029). Among the pregnancy and birth related factors, the presence of any complications during a woman’s pregnancy or birth was associated with increased odds of mistreatment. Women who faced complications were 78% more likely to experience mistreatment compared to those who did not encounter any complications (AOR = 1.78, 95%CI: [1.05 – 3.01], *p*–value = 0.031).

Previous history of stillbirth, mode of birth, and presence of a companion were associated with reduced occurrence of interpersonal abuses. Women with a prior stillbirth were 51% less likely to experience mistreatment (AOR = 0.49, 95%CI: [0.24 – 0.97]; *p*-value = 0.041). Similarly, women who had a caesarean section were 41% less likely to experience mistreatment compared to women who gave birth vaginally (AOR = 0.59, 95% CI: [0.36 – 0.98], *p*-value = 0.042). Furthermore, women who were allowed to have a companion of their choice during maternity care had 42% decreased odds of experiencing mistreatment compared to women who were not permitted (AOR = 0.58, 95%CI: [0.42 – 0.81]; *p*–value = 0.001) (Table 4).

### Association between mistreatment of women and the maternal continuum of care

The association of mistreatment of women with the completion of maternal continuum of care was assessed while controlling for other variables, as presented in Table 5. In this context, completion of the continuum of care involves to meeting specific criteria specified in the methods section, including a minimum of four antenatal care check-ups, childbirth attended by skilled providers, and at least two postnatal care check-ups within the first week after birth. The final model yielded ICC of 25.4%, indicating that 25% of the variability in completing the continuum of care was attributed to the variation in health facilities.

**Table 5:** Association between mistreatment of women and continuum of maternity care in East Wollega Zone, Western Ethiopia, 2022: A mixed-effects binary logistic regression analysis (*n* = 752)

Variables	Null model AOR [95%CI]	Model II AOR [95%CI]	Model III	
			AOR [95%CI]	*P*-values
A. Fixed effects				
(Intercept)	0.04[0.01 – 0.19]	0.05[0.01 – 0.27]	0.00[0.00 – 0.04]	< 0.001
Any interpersonal abuse				
Not experienced		*Reference*	*Reference*	
Experienced		0.49[0.30 – 0.80]	0.49[0.29 – 0.83]	0.008
Poor rapport between woman and healthcare providers				
Not experienced	-	*Reference*	*Reference*	
Experienced	-	0.70[0.44 – 1.10]	0.67[0.42 – 1.08]	0.101
Woman’s age (years)	-	-	1.05[0.99 – 1.11]	0.098
Educational status				
Secondary school and below	-	*-*	*Reference*	
College and above		-	1.77[1.01 – 3.10]	0.046
Income				
Lowest	-	-	*Reference*	
Low	-	-	0.60[0.27 – 1.35]	0.220
Middle	-	-	1.01[0.50 – 2.03]	0.991
High	-	-	1.36[0.64 – 2.86]	0.425
Presence of complication				
No	-	-	*Reference*	
Yes	-	-	3.20[1.73– 5.94]	< 0.001
Quality of antenatal care	-	-	1.25[1.03 – 1.51]	0.023
B. Random effects				
Health facilities				
Variance	1.94	2.31	1.12	-
Intraclass correlation (ICC)	0.370	0.412	0.254	-
C. Model fitness				
AIC	557	547	521	-
BIC	566	566	572	-
Log-Likelihood	-276	-269	-250	-
Deviance	553	539	499	-
*P*-value	-	0.002	< 0.001	-

Women who encountered physical abuse, verbal abuse, stigma, or discrimination during maternity care were found to have a significantly decreased likelihood of completing the continuum of care, with their odds reduced by half compared to those who did not experience such interpersonal abuse (AOR = 0.49, 95% CI: [0.29 – 0.83], *p*-value = 0.008). Although not statistically significant, the presence of poor rapport was also observed to be associated with reduced odds of completing the maternal continuum of care.

Among the social determinants of health, higher education attainment, specifically attending college and above, was found to significantly enhance the likelihood of completing continuum of maternity care. Women with a college education or higher were 77% more likely to complete the continuum of care compared to those with an educational status below college level (AOR = 1.77, 95% CI: [1.01 – 3.10], *p*-value = 0.046). Additionally, the presence of complications during pregnancy or birth also emerged as another factor associated with completing the continuum of maternity care. Women who experienced pregnancy complications were three times more likely to complete the continuum of care than those without complications (AOR = 3.20, 95% CI: [1.73 – 5.94], *p*-value < 0.001). Additionally, receiving a better-quality antenatal care during pregnancy was associated with a 25% increased odds of completing the continuum of maternity care (AOR = 1.25, 95% CI: [1.03 – 1.51], *p*-value = 0.023).

## Discussion

In this study, we report on the prevalence of mistreatment of women during maternity care, factors associated with interpersonal abuse and also the relationship between mistreatment of women and continuum of maternity care. More than a third of women encountered at least one instance of physical abuse, verbal abuse, stigma, or discrimination, with several factors associated with its occurrences. Nearly one-third of women did not receive care that met professional standards, and every other woman reported poor rapport with health care providers. Our study also suggests women were less likely to attend and finish the continuum of care if they endured mistreatment in their initial period of antenatal care or thereafter.

Previous studies have highlighted significant variation in the prevalence of mistreatment experienced by women during maternity care worldwide, ranging from low rates in some countries [12] to nearly universal experiences in others [13]. This study also demonstrates the significant problem of mistreatment, with varying magnitudes observed for different types of mistreatments. Ensuring care meets professional standards and upholds dignity, respect, confidentiality, and involving clients in the decision-making process are crucial for improving the quality of care, as outlined in WHO’s *standards for improving quality of maternal and newborn care in health facilities* [42]. However, the findings from this study reveals a substantial proportion of women experienced care that failed to meet these standards, along with experiencing nuanced interpersonal abuses, poor rapport with healthcare providers and health system related constrains. Identification of these varied categories of mistreatment in current study signifies the need to understand mistreatment of women as a product of complex personal, structural and system issues that need system-wide solutions [42–44].

The occurrence of mistreatment during maternity care was associated with various characteristics of women, such as social status, education, and socioeconomic background, which have been documented to play a significant role [22, 45]. Our study confirms those findings and also identified that women from the lowest income group had higher odds of experiencing interpersonal abuses. This highlights the inequality in care provision for marginalised and impoverished women [46], perpetuating mistreatment as both health care providers and women themselves normalise it. Constraints within the health care system and unequal power dynamics among providers hinder addressing these issues across the continuum of care [47].

Furthermore, women with complications during pregnancy or birth faced intensified interpersonal abuse, emphasising the greater vulnerability of those in need of additional physical and emotional care. Women who had a vaginal birth also faced higher odds of mistreatment compared to women who required a caesarean section, likely due to the increased pain and challenges of spontaneous labour, as reported elsewhere [48]. Conversely, women who had a companion present had a lower risk of experiencing interpersonal abuse, underscoring the positive influence of having a supportive companion present during childbirth [49]. These factors become more pressing in hospitals with higher patient ratios compared to health centres [22, 47], as women tend to bypass health centres, contributing to hospital congestion for various reasons, including perceptions of poor quality of care and infrastructure issues [47].

The occurrence of mistreatment in healthcare facilities undermines service utilisation, contributing to the discontinuation of completing continuum of care [29, 30]. In our study, we found that half of the women who experienced physical abuse, verbal abuse, stigma, or discrimination discontinued maternity care. Women who have experienced mistreatment in any form during pregnancy may be more likely to discontinue prenatal care, even if they had initially intended to give birth at that facility. They prioritise giving birth in a dignified manner and prefer to avoid such mistreatment, despite recognising the potential impact of choosing a home birth. Demonstrating that women are reluctant to seek evaluation and assistance from health care providers who disrespect and abuse them, leading to mistrust, dissatisfaction and even depression [50]. This reluctance contributes to complications and hampers efforts to reduce maternal and neonatal mortalities, thereby impacting on the global sustainable development goals (SDG) target of maternal health [51]. The negative associations between mistreatment and the continuity of care highlight the need for comprehensive, system-wide interventions that go beyond educational measures, such as training, which have been found to be inadequate on their own [51].

Our study presents significant ongoing occurrences of mistreatment of women during maternity care requiring urgent change within the health care system. We acknowledge there are some limitations and strengths in our study. The limitation of this study is that the findings were based on participants only from four districts near the zonal capital city, this was due to challenges in accessing women from remote areas affected by ongoing unrest during data collection in the area. However, efforts were made to include women from rural areas who sought care in nearest health centre and hospital. We believe the occurrence of mistreatment and its effect would not differ significantly in the remaining district and primary hospitals from what observed in current study.

Another important limitation to consider in this study is that we recruited women during immunization due to community unrest in the area. Current study participants may exhibit a higher health-seeking behaviour than those who did not come for vaccination, which could lead to an underestimation of the influence of mistreatment on the continuity of care. A community-based study could have offered a more accurate depiction of the relationship between mistreatment of women and continuity of care. Although over 97% of women in Ethiopia visit health facilities to have their children vaccinated [33], potential participants may have been lost, and therefore, the findings from this study might not be generalisable to the broader population.

Additionally, the cross-sectional nature of our study design limits our ability to establish a causal association between mistreatment and continuity of care, as we were unable to determine whether experiences of mistreatment during previous pregnancies and births also contributed to the discontinuation of continuity of care or if it was solely influenced by the experiences during the current pregnancy and birth.

A notable strength of our study is the exploration of a previously unexplored aspect, the influences of mistreatment on the continuum of maternity care in Ethiopia. Additionally, we measured mistreatment in various categories, considering its multidimensionality and utilising validated survey tools, instead of reporting the magnitude as a single combined measure.

## Conclusions

Mistreatment of women in maternity care is a pervasive issue that extends beyond labour and birth, encompassing antenatal and postnatal care as well. This mistreatment has significant negative consequences, hindering women's engagement and completion of the continuum of care and resulting in reduced utilisation of maternal healthcare services. Such trends have detrimental effects on overall maternal well-being. It is crucial to identify and address these gaps by avoiding interpersonal abuse, enhancing rapport through attitude and value transformation trainings. Such interventions should align with a system level actions, including enforcing respectful care as a competency of every provider, enhancing health centre functionality, improving the referral system, and influencing communities to demand respectful care. Such changes could promote positive maternal and neonatal health outcomes while fostering trust and respect between women and healthcare providers. Collaborative efforts involving healthcare providers, facilities, communities, and policymakers are necessary to ensure equitable and respectful care for all women, irrespective of their social or medical circumstances.

## Data Availability

The dataset used in this study is available from the corresponding author on reasonable request.
